# Intra-abdominal hypertension/abdominal compartment syndrome of pediatric patients in critical care settings

**DOI:** 10.1515/med-2025-1244

**Published:** 2025-07-17

**Authors:** Vesna G. Marjanovic, Ivana Z. Budic, Maja D. Zecevic, Marija M. Stevic, Dusica M. Simic

**Affiliations:** Clinic for Anesthesiology and Intensive Therapy, University Clinical Center Nis, Medical Faculty, University of Nis, Nis, Serbia; Clinic for Pediatric Surgery, Pediatric Orthopedics and Traumatology, University Clinical Center Nis, Medical faculty, University of Nis, Nis, Serbia; Department of Anesthesiology and Intensive Therapy, University Children's Hospital, Medical Faculty, University of Belgrade, Belgrade, Serbia

**Keywords:** intra-abdominal hypertension, abdominal compartment syndrome, children

## Abstract

**Background:**

Intra-abdominal hypertension (IAH)/abdominal compartment syndrome (ACS) is one of the rarer clinical entities in the pediatric population, carrying a high degree of morbidity and mortality. The focus of this review is on assessing pathophysiological changes of organ systems in pediatric patients with risk factors for the occurrence of IAH/ACS based on the evaluation of diagnostic modalities and therapeutic strategies.

**Methodology:**

A comprehensive literature search of indexed databases was performed, aiming to identify, review, and evaluate published articles on IAH/ACS. The search was focused on studies examining pathophysiology, risk factors, diagnostic approaches, and management strategies.

**Results:**

The main risk factors encompass diminished abdominal wall compliance, increased intraluminal and abdominal contents, and capillary leak/fluid resuscitation. Diagnostic tools include clinical and imaging findings, intra-abdominal pressure (IAP) monitoring, and parameters of tissue perfusion. Therapeutic strategies involve non-surgical and surgical management of IAH/ACS in pediatric patients.

**Conclusion:**

Timely and continuous evaluation of IAP and parameters of tissue perfusion is crucial for the early diagnosis of IAH/ACS and implementing non-surgical procedures, reducing the need for surgical procedures. Future research should focus on the usefulness of advanced non-invasive monitoring technologies and the identification of predictors of increased IAP in the early implementation of personalized therapeutic strategies.

## 1 Introduction

Intra-abdominal hypertension (IAH) and abdominal compartment syndrome (ACS) represent life-threatening clinical conditions. IAH in children is defined as a sustained (continuous) or repeated (oscillatory) pathological elevation in intra-abdominal pressure (IAP) above 10 mmHg, while ACS is defined as IAH associated with new or worsening organ dysfunction [1,2]. The incidence of IAH/ACS in pediatric patients is different. It ranges from 10 to 20% in neonatal intensive care unit (NICU) and pediatric intensive care unit (PICU) to 17–63% in specialized pediatric PICUs that hospitalize patients after liver transplantation [3,4,5]. Therein, the incidence of IAH occurs about two to three times more frequently compared to ACS. Thus, the incidence of IAH was 9 and 12.6%, and ACS was 4% in both studies [3,4]. Regarding the mortality rates in the NICU and PICU, it differs from 40 to 100%. Higher mortality rate was recorded in neonatal patients and patients who did not receive decompressive laparotomy (DL), in comparison to older children who underwent DL [4,6,7,8,9,10]. The reason for the high mortality in children lies in the untimely recognition and rapid progression of IAH into ACS, as well as their inadequate treatment compared to adults [1,3,11,12,13]. That can be explained by their smaller abdominal cavity, inability to increase abdominal volume without a significant increase in IAP, despite their greater elasticity of the abdominal wall compared to adults [14]. This indicates that ACS in children is still poorly understood by pediatric intensivists.

The aim of this review is to give insight on the assessment of pathophysiological changes of organ systems in pediatric patients with risk factors for the IAH/ACS, based on the evaluation of clinical and imaging findings, levels of IAP, abdominal perfusion pressure (APP), near-infrared spectroscopy (NIRS), hemodynamic parameters, and biomarkers. This information can help in earlier diagnosis and implementation of therapeutic procedures, with improvement in the survival of these patients. Due to the limited information associated with the untimely recognition and diagnosis of IAH/ACS in children, many issues addressed in this report can be widely applied in the treatment of critically ill pediatric patients in the PICU.

## 2 Pathophysiological changes in pediatric patients with IAH/ACS

Elevated IAP can cause pathophysiological changes in abdominal and extra-abdominal organs due to direct compression and impaired oxygen delivery to tissues, leading to ischemia, anaerobic metabolism, and lactic acidosis [4]. Usually, the initial increase in IAP is well tolerated in children due to good compliance of their abdominal wall. However, with further elevation of IAP, IAH occurs, which leads to the compression of abdominal blood vessels, splanchnic hypoperfusion, ischemia of the intestines, and enterocyte necrosis. This disrupts the barrier function of the intestinal wall, leading to bacterial translocation, the release of pro-inflammatory cytokines into the systemic circulation, the development of a systemic inflammatory response, and sepsis. Due to altered inflammatory processes and lymphatic drainage in the intestines, intestinal edema develops, which further increases the degree of IAH and leads to the development of ACS [11]. In addition to the pathophysiological changes in the abdomen, changes in the respiratory, cardiovascular, urinary, and neurological systems can occur [15]. Thus, elevated IAP causes diaphragm elevation, increased intrathoracic pressure, and lung tissue compression, which reduces functional residual capacity and tidal volume and leads to atelectasis. These lung changes alter the ventilation-perfusion ratio within the lungs, increase intrapulmonary shunt, and lead to hypoxemia and hypercapnia. Consequently, these patients often require mechanical ventilation, which may prolong their hospitalization in the PICU and increase their mortality [5]. On the other hand, increased intrathoracic pressure reduces preload into the right heart, decreases cardiac output due to compression of the inferior vena cava and portal vein, and increases systemic afterload due to direct compression of the pulmonary and abdominal vascular beds. That can lead to the development of heart failure in pediatric patients even with mild IAH [16,17]. Reduced cardiac output leads to systemic hypoperfusion and rapid multiorgan failure [12]. Renal hypoperfusion arises due to decreased cardiac output and compression of the renal artery and vein. It leads to oliguria as the first sign of progression of IAH to ACS, and later anuria [4]. Reduced liver perfusion in IAH can lead to decreased lactate clearance and glucose metabolism disturbances [4,12]. Finally, increased intrathoracic pressure in IAH is transmitted to the superior vena cava system, impeding venous return from the brain. This can lead to increased intracranial pressure, which is clinically significant in patients with head trauma or brain edema [17].

## 3 Risk factors for the development of IAH/ACS in children

During each hospitalization of pediatric patients in the PICU, it is necessary to screen risk factors for IAH/ACS [1,18]. The main risk factors for increased IAP in children include diminished abdominal wall compliance, increased intraluminal and abdominal contents, and capillary leak and fluid resuscitation [1,12].

### 3.1 Diminished abdominal wall compliance

Diminished abdominal wall compliance can be expected in patients with acute respiratory insufficiency accompanied by high intrathoracic pressure, trauma, or extensive burns (circumferential 3rd degree burns of the abdominal wall), prone positioning, and head elevation higher than 30°, obesity, gastroschisis, omphalocele, diaphragmatic hernia, or closure of the abdominal wall under increased tension.

### 3.2 Increased intraluminal and abdominal contents

The intraluminal content increases with the accumulation of air, fluid, or stool in the intestines in patients with gastroparesis, intussusception, adynamic ileus, pseudo-obstruction of the colon, constipation, or Hirschsprung’s disease. The abdominal content increases with the accumulation of air, fluid, and blood in the abdomen in patients with abdominal trauma, retroperitoneal bleeding, hemoperitoneum and pneumoperitoneum, peritonitis or enterocolitis, splenomegaly and hepatomegaly, intra-abdominal tumors, liver dysfunction with ascites, pancreatitis, kidney, liver, and intestine transplantation, and extracorporeal membrane oxygenation. All these clinical conditions characterized by diminished abdominal wall compliance, increased intraluminal and abdominal contents, are the cause of primary ACS.

### 3.3 Capillary leak/fluid resuscitation

Secondary ACS is defined as a condition that does not originate from the abdominopelvic region but is associated with capillary leak seen during aggressive fluid resuscitation in critically ill patients or in diseases associated with capillary leak syndrome [2]. Thus, aggressive fluid resuscitation with crystalloids increases the risk of ACS in pediatric trauma patients and hemato-oncological patients in the PICU as well as in those with septic, cardiogenic, or toxic shock syndrome, acidosis with pH <7.2, hypotension, hypothermia, massive transfusion, coagulopathy, pancreatitis, oliguria, severe burns, laparotomy for damage control, or SIRS, and after liver or kidney transplantation, or heart transplant rejection [19,20,21].

The most common associated pediatric conditions that predispose patients to ACS are abdominal trauma with intensive bleeding, ileus, necrotizing enterocolitis, significant gas accumulation without occlusive bowel lesions, ascites, diaphragmatic hernia, septic shock followed by massive fluid resuscitation, and multiorgan distress syndrome [4,15,21,22,23,24]. Thereby, pediatric patients with risk factors for IAH/ACS occurrence should be recognized in a timely manner during their admission to the PICU.

## 4 Diagnosis of IAH/ACS

### 4.1 Clinical and imaging findings

The correct diagnosis of ACS can be made based on clinical indicators associated with risk factors and confirmed with a recording of elevated IAP. In this syndrome, a rapid onset of abdominal distension is registered, which limits the capacity of lung expansion and leads to reduced tissue oxygen saturation, refractory hypotension, and oliguria/anuria [25,26]. The syndrome can develop rapidly or in days after the injury, so high clinical suspicion and observation of these patients is of utmost importance, especially patients who have suffered multiple traumas, required assisted mechanical ventilation, or had a history of abdominal surgery or abdominal disease, which are followed with increased IAP [27]. The management of these patients should occur in a PICU, since their high complexity and the list of extensive differential diagnoses. Therefore, daily physical examination and observing parameters such as blood pressure, intake, and excretion measurements are critical to its diagnosis. Methods such as manual palpation of the abdomen and abdominal circumference measurement are insensitive in correlation with increased IAP, which is why it is not recommended. Until now, many clinicians still use this method in everyday clinical practice. However, abdominal distension, a plateau pressure of more than 30 cm H_2_O, and hypothermia have been identified as independent predictors of IAH on the admission day of these patients [3,28]. The most common imaging findings on magnetic resonance and computed tomography are ascites, inferior vena cava compression, heterogeneous perfusion of the kidneys, elevation of diaphragm, basal lung atelectasis, abnormal enhancement of the bowel wall, and subcutaneous edema [29]. The imaging findings were not proved specific for ACS but should raise suspicion for ACS in children.

### 4.2 IAP monitoring

The most accurate method to confirm the diagnosis of IAH/ACS is measuring the IAP [3,4,8,9,30,31]. IAP measurement should be obtained if one or more risk factors for IAH/ACS are present [1,18]. IAP measurement technique can be achieved using direct and indirect methods. The direct measurement of the pressure in the peritoneal cavity is an invasive method, which shows greater accuracy in measuring IAP compared to the intravesical method. This method is conducted by placing a needle or an abdominal catheter into the peritoneal space. It is suggested for patients for whom an abdominal catheter has previously been placed as part of a therapeutic procedure. Complications of this method of measuring IAP include bowel perforation and peritoneal contamination [32].

Indirect measurement is noninvasive and can be measured in different intracorporal cavities such as the stomach, rectum, uterus, and bladder. Indirect estimation of IAP taken from the bladder cavity is the gold standard and is recommended by the Abdominal Compartment Society [1,4,18,31,32,33]. It is performed after bladder catheterization with a Foley catheter and connecting the Foley to a three-way stopcock adjusted to the level of the mid-axillary line at the iliac crest to zero the transducer. The sterile saline in volume 1 ml/kg to up to a maximum of 25 ml is then injected into the bladder. The measurement of IAP should be taken with the patient in a supine position and at end-expiration [1]. The most common reasons for errors in implementing this method are the presence of air bubbles in the system, incorrect positioning of the pressure transducer, and obstruction of the urinary catheter. When instilling a larger volume of fluid into the urinary bladder than recommended, falsely elevated IAP values are obtained, which can lead to unnecessary therapeutic procedures [31,34]. Prolonged use of a standard latex urinary catheter and continuous measurement of IAP can increase the risk of urinary infection [21]. However, there are catheters for continuous monitoring of IAP (Accuryn, for example) that do not have a higher risk of catheter-associated urinary tract Infection. This could be an area to be explored in future research, as continuous real-time monitoring of IAP has obvious advantages in critically ill children.

In healthy, spontaneously breathing children, IAP is often around 0 mmHg. In mechanically ventilated and critically ill children, IAP is approximately 7 ± 3 mmHg and 4–10 mmHg, regardless of the child’s body weight [8,21,34]. It should be noted that the criteria for IAH and ACS in children and adults are different. In children, IAH is defined as IAP above 10 mmHg, while ACS is defined as IAP greater than 10 mmHg associated with new or worsening organ dysfunction/failure. In adults, IAH is defined as IAP above 12 mmHg, while ACS is defined as IAP greater than 20 mmHg with evidence of new or worsening organ dysfunction/failure [1,30] (Table 1).

**Table 1:** Comparison of definition IAH/ACS in pediatric patients and adults

	Children	Adults
Normal IAP	4–10 mmHg in critically ill children	Approximately 5–7 mmHg in critically ill adults
IAH	A sustained or repeated pathological elevation in IAP ≥10 mmHg	A sustained or repeated pathological elevation in IAP ≥12 mmHg
IAH grade I	IAP 10–12 mmHg	IAP 12–15 mmHg
IAH grade II	IAP 13–15 mmHg	IAP 16–20 mmHg
IAH grade III	IAP 16–19 mmHg	IAP 21–25 mmHg
IAH grade IV	IAP ≥20 mmHg	IAP >25 mmHg
ACS	A sustained IAP >10 mmHg associated with new organ dysfunction/failure	A sustained IAP >20 mmHg that is associated with new organ dysfunction/failure

IAP = intra-abdominal pressure; IAH = intra-abdominal hypertension; ACS = abdominal compartment syndrome.

Note: Data from Kirkpatrick et al. [1], Cheatham et al. [30], and Ejike et al. [34].

That means that a small increase in IAP may be sufficient to compromise abdominal perfusion and cause the ACS in children for a short time [1,17,30,35]. The reason is that mean arterial pressure (MAP) is naturally lower in pediatric patients [36]. Therefore, it is recommended that pressure measurements should be performed every 6 h in patients with IAH and with potential for development of ACS [1,2,3,4,9,30,31].

In addition to the bladder measurement of IAP, intragastric pressure can be measured. The measurement of intragastric pressure is carried out by placing a nasogastric tube and an air capsule, which enables continuous measurement of IAP. It shows a high correlation with bladder measurement of IAP, thereby improving the identification of critically ill children with suspected ACS [37].

### 4.3 Parameters of tissue perfusion

Other parameters, which can be used for assessment of tissue perfusion in IAH/ACS, are APP, NIRS, hemodynamic parameters, and biomarkers.

APP has previously been suggested as a more accurate predictor of visceral perfusion and a better endpoint for resuscitation than IAP or MAP alone [2]. This value is calculated using the formula APP = MAP − IAP. According to the Abdominal Compartment Society, in adults and children older than 5 years, the resuscitation goal for APP should be a cut-off value of 50–60 mmHg [1]. However, APP values in children younger than 5 years are significantly lower, especially in infants, where the critical cut-off is as low as 35 mmHg. Lower APP values may be explained by a physiologically lower MAP in these age groups [5,20,38]. Until now, there have been no recommendations for APP in pediatric patients. Therefore, there is a need for studies aiming to identify critical cut-off APP values for all age groups.

NIRS is a noninvasive technique for the measurement of regional tissue oxygenation and assessment of microcirculation. This technique is used in monitoring abdominal, renal, or cerebral perfusion [39,40,41,42]. Thereby, this technique has shown usefulness in distinguishing healthy from ischemic intestines and detecting decreased renal and cerebral perfusion during and after the primary or gradual abdominal closure in neonates with gastroschisis [43,44]. Gradual abdominal wall closure of gastroschisis is not recommended in neonates with a specific cut-off NIRS level below 42% and cerebral splanchnic oxygenation ratio below 0.76 due to the risk of intestinal ischemia [45]. Although this technique may provide evidence about cerebral and renal regional oxygen saturation, determination of the mesenteric regional tissue oxygenation is considered an appropriate parameter in predicting IAH and poor clinical outcome of critically ill children [46]. So, a critical cut-off value for mesenteric tissue oxygenation below 50% was predictive for detecting IAH in children. Although this parameter was monitored noninvasive and continuously, determination of its cut-off value was performed only at 6-h intervals [46]. In addition, in the study of Junge et al. [5], no association was found between changes in NIRS and the presence of IAH or ACS due to the different weight and ages of the patients. Due to inconsistent data, further studies to determine critical cut-off values for NIRS for each age group during a 24 h interval are needed.

In addition to NIRS, by determining the values of mixed venous oxygen saturation, vasoactive inotropic score, and degree of distension of the left internal jugular vein, more precise data on the degree of negative hemodynamic changes in pediatric patients with IAH are obtained [17,46]. Biomarkers, such as level of lactate, intestinal fatty acid-binding protein in plasma, vascular endothelial growth factor (VEGF), π-glutathione S-transferase, and monocyte chemoattractant protein in urine, VEGF, and creatinine in serum, may be helpful in noninvasive assessment of organ disfunction in children with ACS [27,47]. Of all the above, only lactate levels represent an independent predictor of IAH presence [12,27]. Until now, hemodynamic parameters and biomarkers except lactate have not shown reliable clinical evidence in predicting IAH/ACS and therefore may be an area of additional research in the future.

Clinical and imaging findings with measurement of IAP, APP, NIRS, hemodynamic parameters, and biomarkers may be helpful in determining the degree of organ damage in patients with IAH/ACS. Considering that lower values of IAP in children for a short time can lead to ACS in comparison with adults, continuous evaluation of these parameters may be crucial for early diagnosis and prompt implementation of therapeutic procedures.

## 5 Therapeutic strategies concerning the management of IAH/ACS

The strategy for optimal treatment involves a set of appropriate interventions aimed at reducing IAP prior to its progression to compartment syndrome and the need for invasive treatment such as DL in these patients [1,11,30]. Timely medical suspicion is essential to initiate therapeutic measures to avoid organ dysfunction and death. Prior to the start of treatment, the search for the etiology represents a cornerstone because it determines the most appropriate therapeutic strategy [11]. There are well-described non-surgical and surgical strategies for effectively reducing IAP in pediatric patients with IAH/ACS. Summary of risk factors, diagnostic modalities, and treatment strategies are given in Table 2.

**Table 2:** A summary of risk factors, diagnostic modalities, and treatment strategies

Risk factors	Diagnostic modalities	Treatment strategies in IAH/ACS
Diminished abdominal wall compliance	Clinical and imaging finding	Improving abdominal wall compliance
	IAP monitoring	Deep sedation, analgesics, NMBs, reverse Trendelenburg position
	Parameters of tissue perfusion	Surgical decompression
Increased intraluminal contents	Clinical and imaging finding	Evacuation of intra-luminal content
	IAP monitoring	Gastric decompression with NGT, prokinetics, enemas, rectal tube, stopping or minimizing EN
	Parameters of tissue perfusion	Surgical decompression
Increased abdominal contents	Clinical and imaging finding	Evacuation of intra-abdominal content
	IAP monitoring	Surgical decompression
	Parameters of tissue perfusion	
Capillary leak/fluid resuscitation	Clinical and imaging finding	Optimizing fluid administration, and systemic perfusion
	IAP monitoring	Goal-directed fluid resuscitation (negative fluid balance in hypervolemia), hemodialysis, vasoactive drugs
	Parameters of tissue perfusion	Surgical decompression

IAH = intra-abdominal hypertension; ACS = abdominal compartment syndrome; IAP = intra-abdominal pressure; NMB = neuromuscular blocker; NGT = nasogastric tube; EN = enteral nutrition.

Note: Data from Kirkpatrick et al. [1], Liang et al. [15], and Gottlieb et al. [18].

### 5.1 Non-surgical strategy of IAH/ACS

Non-surgical strategy involves numerous medical and minimally invasive therapies that are indicated in pediatric patients with mild to moderate IAH [1]. This strategy should always be applied as early as possible, reducing the need for surgical interventions [31]. Non-surgical strategies include evacuation of intra-luminal content, improving abdominal wall compliance, evacuation of intra-abdominal space-occupying material, optimizing fluid administration, and systemic perfusion.

Evacuation of intra-luminal contents involves the use of a nasogastric tube, prokinetics, enemas, and rectal tube, as well as stopping or minimizing enteral feeding in these patients [1]. Improving abdominal wall compliance may decrease excessive abdominal muscle contraction, ensure comfort, and protect the intestinal function of patients with mild-to-moderate IAH [1,18]. This may be achieved by using sedatives, analgesics, neuromuscular blockers, and body positioning of patients in the reverse Trendelenburg position. Both measures may be effective only for the short term in the reduction of IAP [1,5,11]. The effectiveness of such measures also depends on etiology since patients with extraluminal collections will not respond to the mentioned measures but will respond to percutaneous peritoneal drainage [15]. Optimizing fluid administration and systemic perfusion involves goal-directed fluid resuscitation, correcting positive fluid balance, and hemodialysis. In the study published by Kirkpatrick et al. [1], goal-directed fluid resuscitation implies avoiding large volumes of crystalloids and early use of hypertonic and colloid solutions after completing the acute resuscitation phase. This procedure and hemodialysis aim to achieve a net negative or zero fluid balance in patients with IAH to reduce the progression of secondary ACS [3]. In addition, the administration of vasoactive drugs and hemodynamic monitoring are often crucial in ensuring an adequate level of APP [1,31]. These suggestions of the author cannot be recommended yet. However, the current Abdominal Compartment Society guidelines do not recommend the use of APP in the resuscitation process [1]. The reason for that lies in the fact that elevated MAP, resulting from increased systemic vascular resistance and decreased cardiac output, can lead to hypoperfusion despite an acceptable APP. Therefore, optimizing fluid administration and systemic perfusion remains a challenge for clinicians.

Clinicians should not overestimate non-surgical treatment measures in their effectiveness. If the above measures fail, surgical decompression using emergent laparotomy should be considered.

### 5.2 Surgical strategy of IAH/ACS

Surgical management of ACS includes percutaneous catheter drainage (PCD), and DL, which may decrease IAP and improve organ function and outcome of these patients [15,18,48]. PCD is a popular method of extraluminal volume reduction caused by the accumulation of air, fluid, or blood in the abdominal cavity. Because of its less invasiveness, PCD may alleviate the need for more invasive methods or provide the time needed to stabilize patients before DL is implemented [49,50]. It is most used in neonates with necrotizing enterocolitis, in patients with massive ascites, burns, IAH grade III or IAH with some organic dysfunction, and when conservative measures are insufficient [15,36,50,51]. After PCD, the level of IAP, degree of abdominal distension, and organic dysfunction are significantly reduced with a simultaneous increase of MAP, APP, glomerular filtration rate, urine output of >2 mL/kg/h, and PaO_2_/FiO_2_ ratio [36,49,50]. In most cases, IAP usually normalizes within 24 h, and the abdominal catheter can be removed. Potential complications of this method include abdominal infection and electrolyte imbalances in these patients [15]. With this management, the mortality rate is approximately 25%, and organic improvement was observed [1,15].

As a last alternative, surgical decompression should be considered. DL is an invasive and effective surgical measure to treat the most severe form of ACS in children. Implementation of this method results in an immediate decrease in IAP and improvements in organ function [4,8,25,36,48,52,53,54,55]. In the studies of Rezeni and Thabet [31] and Laconi et al. [56], the main criteria for DL were a rapid increase in abdominal diameter of 1 cm within 24 h, increased ventilatory support after 8–12 h from the rise in IAP, reduced diuresis below 1 ml/kg/h, and worsening NIRS after 6–8 h from the increase in IAP. In the studies of Pearson et al. [48] and Van Damme and De Waele [54], the main criteria for surgical decompression were the deterioration of ventilatory parameters and the need for mechanical ventilation with higher ventilation pressures, persistent oliguria, and serum lactate greater than 3 mg/dL. The secondary criteria for surgical decompression were hypertension, coagulation disorders, collateral circulation, and the need for vasopressor support. Combining two main criteria or one main and three secondary criteria has been used to indicate surgical decompression [44,57]. DL may be the most effective when performed within 8 h of the onset of ACS [58]. Thus, early decision-making for DL should be encouraged, as it can save children’s lives [59]. The degree of reduction of IAP with this method varies depending on the study. In the study by Divarci et al. [4], IAP was reduced from 20 to 9 mmHg. In another study by di Natale et al. [52], IAP was reduced from 22.5 to 9 mmHg, with a simultaneous improvement in APP from 23 to 44.5 mmHg. Significant reduction of IAP and improvement of organ perfusion are expected within 6–12 h after DL [4,8,25,33,35,48,52,53,54,55]. DL is associated with overall patient mortality of over 50%, especially with children under 1 year of age, weight under the third percentile, an open abdomen treatment, an intestinal resection, and an elevated serum lactate >1.8 mmol/L. Late implementation of this procedure exhibits a higher mortality rate and its limiting effect [52,60,61]. Until now, different criteria and timing have been used for the implementation of DL in pediatric patients with ACS, which has resulted in various reductions of IAP. New studies in the future should more precisely define the criteria and time of DL application, which will help to improve the efficiency of this method and reduce the mortality of children with ACS.

This technique can be handled with full closure or associated with a temporary abdominal closure (TAC). TAC is a reliable and safe method in treating severely injured and acute care surgery patients. The different techniques account for different results according to the different indications. In peritonitis, commercial negative pressure temporary closure seems to be the most effective in reducing mortality. In trauma, skin closure and Bogotà bag seem to provide better results than negative pressure ones. This can be partially explained by the relative absence of infection and cytokines to be cleared [62,63]. Despite the weakness of the literature and the lack of consensus regarding the use of commercial negative pressure temporary closure, it may be used in neonates with necrotizing enterocolitis, patients with peritonitis, and burn patients [64,65]. The degree of negative pressure has to be continuous, with a pressure of −50 to −75 mmHg for children younger than 2 years and −75 to −125 mmHg for children over 2 years of age [66]. The only potential complication associated with its use is the development of enterocutaneous fistulas [67]. The possible causal relationship between negative pressure temporary closure and the development of enteric fistula is still unclear. One of the reasons is that using the same vacuum-assisted closure device for both adults and children is not acceptable, despite the need to adjust the parameters to meet the needs of this population. Therefore, attention should be focused on protecting the delicate tissues and achieving the appropriate level of negative pressure in accordance with the patient’s age. Regardless of complications, this technique has significantly reduced the previously observed morbidity associated with open abdomens and improved patient survival to 80% [67,68]. Further research will be able to define the indications and benefits of this method, determining where this method can be applied in pediatrics [69]. A summary of various criteria for DL is given in Table 3.

**Table 3:** A summary of various criteria for decompressive laparotomy

No	Study	Year	Criteria for decompressive laparotomy
1	Pearson et al. [48]	2010	IAH (range, 12–44 mmHg)
			Maximum ventilatory support
			Required vasopressors/inotropes
			Oliguria and hemodialysis
2	Divarci et al. [4]	2016	IAH
			Increased RR
			Low APP
			Oliguria
3	Van Damme and De Waele [54]	2018	IAH (mean IAP 20.7 mmHg)
			Increased PIP and lower P/F ratio
			Lower MAP and increased CVP
			Oliguria
4	Laconi et al. [56]	2022	IAH
			Abdominal diameter increased >1 cm in 24 h
			Increase of ventilatory support in 8–12 h
			NIRS parameters worsening in 6–8 h
			Oliguria
5	Rezeni and Thabet [31]	2022	IAH
			Abdominal distension
			Increased ventilator setting and oxygen requirement
			Increased vasopressor or inotrope dose
			Oliguria
			Worsening acidosis

IAH = intra-abdominal hypertension; RR = respiratory rate; APP = abdominal perfusion pressure; PIP = peak inspiratory pressure; P/F ratio = ratio of partial pressure of arterial oxygen and fraction of inspired oxygen; MAP = mean arterial pressure; CVP = central vein pressure; NIRS = near-infrared spectroscopy.

Therefore, the early application of non-surgical therapeutic procedures can reduce IAP, decrease the need for surgical therapeutic procedures, and improve the patient’s survival. The final decision on when to convert from non-surgical to surgical therapeutic procedures will be made by pediatric intensivists and surgeons in their interdisciplinary communication based on an assessment of clinical and imaging findings, levels of IAP, APP, NIRS, hemodynamic parameters, and biomarkers in pediatric patients. Early involvement of surgeons and emergency DL is often imperative for these patients.

## 6 Conclusion

ACS rarely occurs in pediatric patients but is associated with high mortality. Clinicians should screen pediatric patients in the intensive care unit with the risk factors, clinical and imaging indicators for the development of ACS. ACS in children must be recognized early because its development needs a lower level of IAP. Timely and continuous evaluation of IAP, APP, NIRS, hemodynamic parameters, and biomarkers will speed up the accurate diagnosis of ACS and the clinician’s decision about the treatment options. Implementation of non-surgical therapeutic procedures will improve organ function and decrease the need for surgical interventions. Current therapies, such as abdominal decompression, appear to positively impact patient survival. However, prospective randomized studies are needed to define advanced non-invasive monitoring technologies and predictors of increased IAP before clinicians decide to replace non-surgical with surgical therapeutic procedures. That will help clinicians in the early implementation of personalized surgical therapeutic options and improve survival in children.

## Abbreviations

IAH: Intra-abdominal hypertension
ACS: Abdominal compartment syndrome
IAP: Intra-abdominal pressure
APP: Abdominal perfusion pressure
NICU: Neonatal intensive care units
PICU: Pediatric intensive care units
NIRS: Near-infrared spectroscopy
MODS: Multiorgan distress syndrome
VEGF: Vascular endothelial growth factor
MV: Mechanical ventilation
BP: Blood pressure
SpO_2_
: Tissue oxygen saturation
MRI: Magnetic resonance imaging
CT: Computerized tomography
NMBs: Neuromuscular blockers
NGT: Nasogastric tube
EN: Enteral nutrition
PCD: Percutaneous catheter drainage
DL: Decompressive laparotomy
TAC: Temporary abdominal closure
